# Cantharidin and Its Anhydride-Modified Derivatives: Relation of Structure to Insecticidal Activity

**DOI:** 10.3390/ijms14010001

**Published:** 2012-12-20

**Authors:** Wenbo Sun, Zhongyi Liu, Yalin Zhang

**Affiliations:** Key Laboratory of Plant Protection Resources & Pest Management of Ministry of Education, Northwest A & F University, Yangling 712100, Shaanxi, China; E-Mails: wbsun06@yahoo.com.cn (W.S.); zhy.liu@hotmail.com (Z.L.)

**Keywords:** cantharidin, anhydride ring opening, derivatives, synthesis, larvicidal activity, structure-activity relationship

## Abstract

Cantharidin is a natural compound of novel structure with ideal insecticidal activity. However, the relationship of structure to insecticidal activity of cantharidin and its derivatives has not been ever clarified. To explore what determines the insecticidal activity structurally of cantharidin-related compounds, two series target compounds **6** and **7** were synthesized by replacing the anhydride ring of norcantharidin with an aromatic amine or fatty amine with different electron density, respectively. The structures of these compounds were characterized by ^1^H NMR, ^13^C NMR and HRMS-ESI. A bioassay showed that compounds **6** (**a**–**m**) lacked any larvicidal activity against *Plutella xylostella*; whereas their ring-opened partners **7** (**a**–**m**) provided a variety of larvicidal activities against *P. xylostella*, and compound **7f** indicated the highest larvicidal activity with LC_50_ value of 0.43 mM. The present work demonstrated that the form of the compound (cyclic or ring-opened) or their ability to hydrolyze facilely was the key to determine whether it exhibits larvicidal activity. Moreover, it revealed that the improvement of insecticidal activity required a reasonable combination of both aliphatic amide and aromatic amide moieties, and the type of substituent Y on the aniline ring was critical.

## 1. Introduction

**Figure 1:**
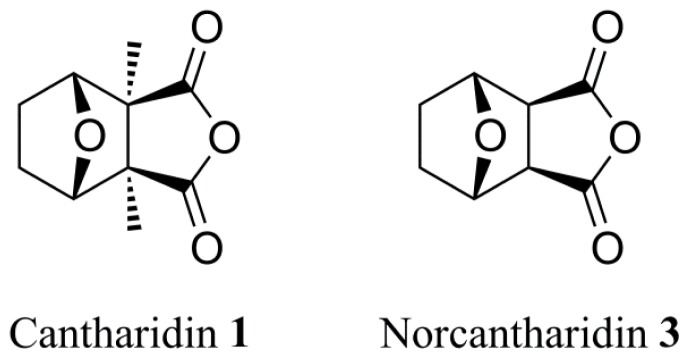
The structures of cantharidin **1** and norcantharidin **3**.

The blister beetle *Mylabris phalerata* (Coleoptera: Meloidae) has been used for centuries as a traditional Chinese medicine. Cantharidin **1** (Figure 1), the active ingredient in the blister beetle toxin, has served as a valuable drug against several kinds of cancer. Similar to what occurs when a variety of human cancer cell lines are co-cultured with cantharidin, insect cell lines Spex-VII and Sf9 can be induced to undergo apoptosis by this toxin [1–4]. Among the novel applications of cantharidin and its analogs were preliminary studies of the anti-insect activity of this natural product.

Cantharidin has been confirmed to be toxic to arthropods in a diversity of orders: Coleoptera, Diptera, Hymenoptera, Homoptera, Lepidoptera and Acarina [5–7]. Apoptosis-like symptoms of insect tissues (midgut, Malpighian tubules, testes, fat body and epidermis) due to cantharidin poisoning of insects were observed under the transmission electron microscope [8–10]. Additionally, the activities of acid phosphatase, alkaline phosphatase and polyphenol oxidase in cantharidin-treated *Mythimna separata* significantly declined [11].

Whereas the procedure for synthesizing cantharidin under assay conditions is complex and difficult [12–14], its analogs are much easier to be synthesized and are promising substitutes for cantharidin. However, cantharidin derivatives possess different structures and are likely to have either higher or lower insecticidal effects.

Our study is designed to explore the relationships between the structures and insecticidal activities of cantharidin analogs. Two series of anhydride-modified cantharidin analogs were designed and synthesized with norcantharidin **3** (Figure 1) as the parent compound. Among them, 6b, 6g, 6m, 7a, 7b, 7e and 7f, seven derivatives, were new compounds. Their insecticidal activities were evaluated against the pre-third-instar larvae of *Plutella xylostella*, *in vivo*.

## 2. Results and Discussion

### 2.1. Chemistry

**Scheme 1:**
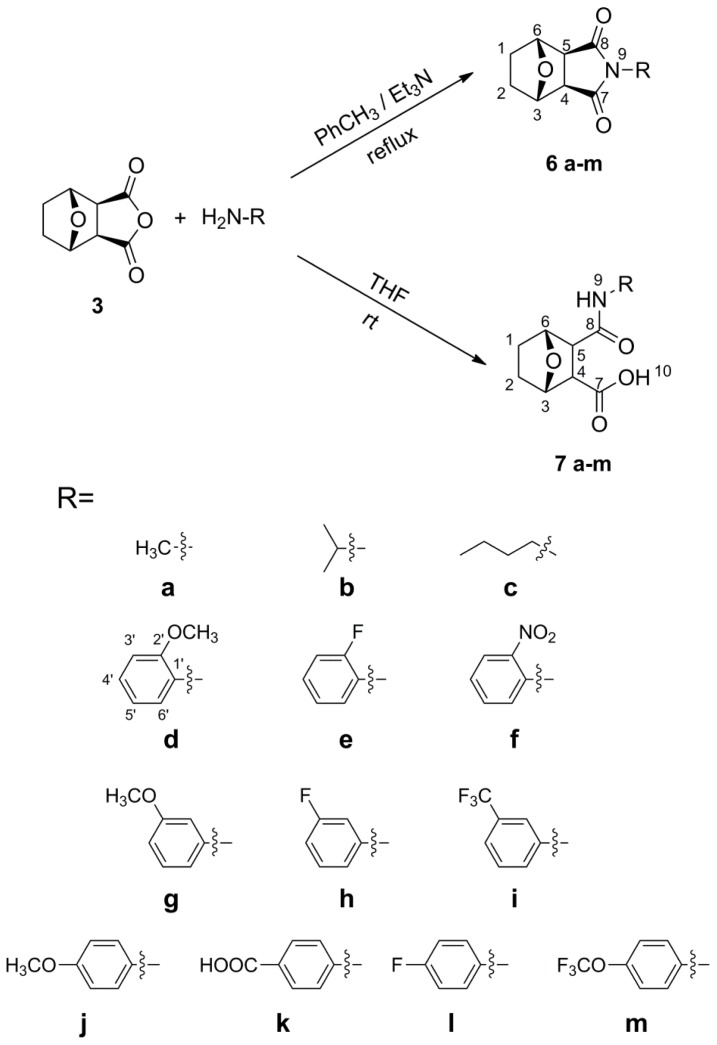
The structure and synthesis of compounds **6** and **7**.

As shown in Scheme 1, the target analogs **6** were synthesized by the reaction of norcantharidin **3** and various primary amines in the presence of triethylamine as the binding acid agent [15], and the analogues **7** were synthesized by replacing the anhydride ring of norcantharidin **3** with an aromatic amine or fatty amine of different electron density [16].

**Scheme 2:**
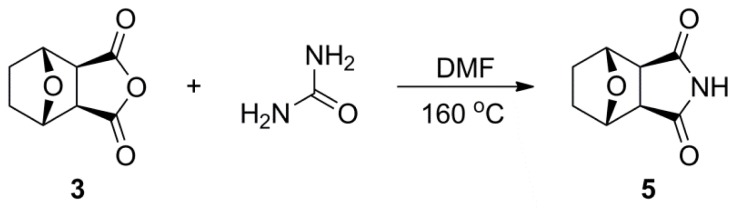
The structure and synthesis of compound **5**.

Compound **5** was synthesized to imitate the synthetic procedure of phthalimide [17], as shown in Scheme 2.

**Scheme 3:**
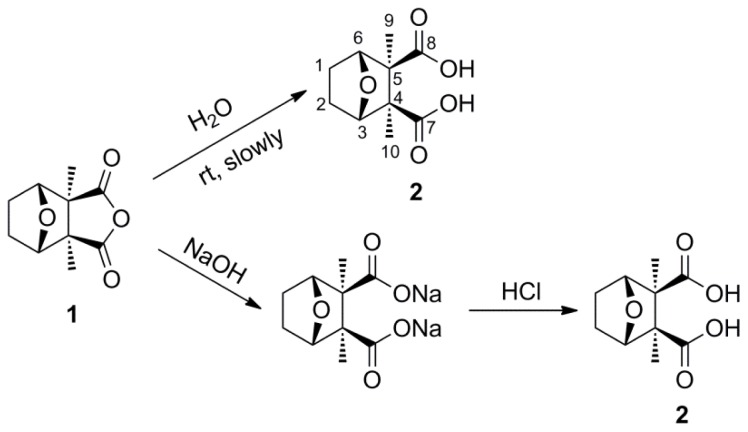
The structure and synthesis of compound **2**.

**Scheme 4:**
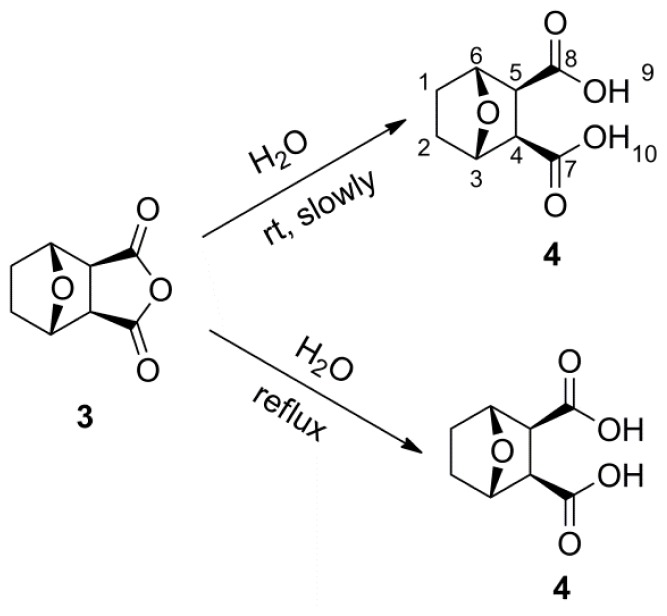
The structure and synthesis of compound **4**.

As can be seen from Schemes 3 and 4, compounds **1** and **3** underwent a rapid conversion to dicarboxylic acid (compounds **2** and **4**) under assay conditions, or through the slow but facile way that the hydrolyzing occurred at room temperature.

Thus the hydrolytic stabilities of analogs **6** presented in Table 1 were examined. We noted that analogs **6** were stable under assay conditions, showing no decomposition even under infrared radiation (typically no decomposition after 12 h treatment).

### 2.2. Insecticidal Activity

After a period of starvation, released larvae wriggled around and attempted to eat the nearby treated leaves. They would not present a series of symptoms until a certain amount of the drug was taken. All active compounds caused the same symptoms.

**Figure 2:**
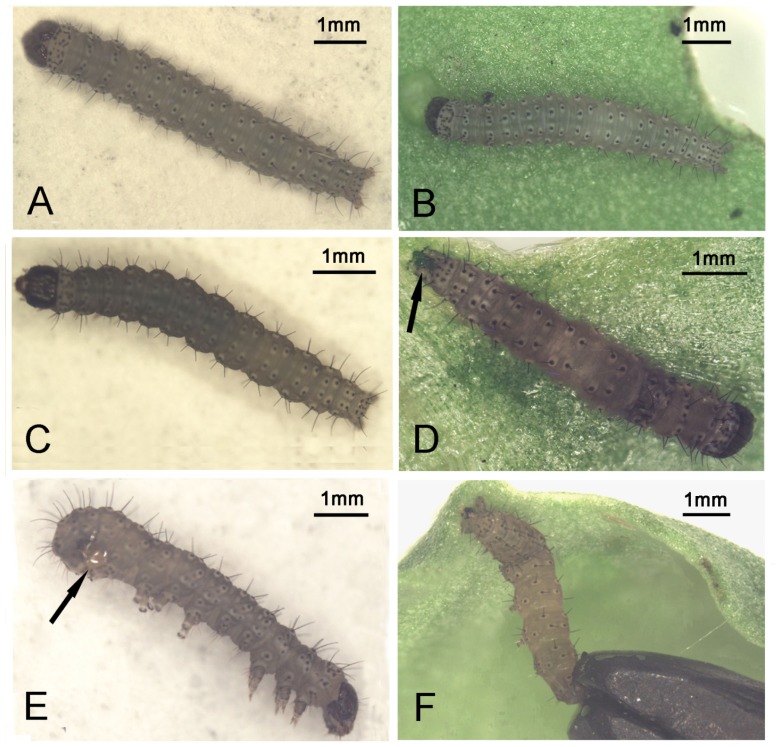
(**A**, **B**) Healthy larvae with light and uniform body color; (**C**) A darker patch appeared anteriorly on a dying larva poisoned by cantharidin; (**D**) Darker patches spread all over the body of a dead larva poisoned by cantharidin with wet, green frass stuck to its anal area, as shown at the arrow; (**E**) Mucus was kept between the fourth pair of prolegs and caudal prolegs as shown at the arrow; (**F**) A larva that died from cantharidin was glued posteriorly and ventrally to a leaf by mucus.

Unlike most commercial pesticides currently used, the poisoned larvae gradually lost their locomotive ability and were powerless to resist mechanical irritations. Meanwhile, compared with the healthy larvae (Figure 2A,B), a darker patch often appeared anteriorly on each dying larva (Figure 2C), which would spread gradually to the whole bodies until they died (Figure 2D). Wet, green frass might stick to the anal areas of dying larvae (Figure 2D), and mucus was frequently observed at the end of the abdomen of dead larvae, which either was kept between the fourth pair of prolegs and caudal prolegs (Figure 2E), or it glued dying larvae to leaves or filter papers (Figure 2F) indicating the experimental drugs might disrupt the digestive system in larvae.

In addition, cantharidin is well known as a strong inhibitor of serine/threonine protein phosphatases (PPs) [18], a broad class of PPs associated with signaling and control of numerous cellular processes in many organisms [19]. The toxicity associated with cantharidin is derived from its ability to inhibit the family of PPs. However, the catalytic domain of all PP subfamilies is highly conserved in animals, plants, protozoans and all eukaryotes [20]. Inhibitors, such as cantharidin and norcantharidin, bind to a hydrophobic pocket of the PP active site [21]. The structural similarity between cantharidin and norcantharidin has been apparent to animal scientists and similar mechanisms of action on animal serine/threonine PPs have been confirmed [22]. Like all other eukaryotes, the family of serine/threonine protein phosphatases belonging to insects and related arthropods should be considered since it is highly similar to that of mammals. Hence, it is deduced that PPs are the potential target sites of insecticidal activity of cantharidin and its related compounds. Meanwhile, because of the high conservation of the catalytic domain of all PP subfamilies, cantharidin and its derivatives are indeed toxic to all eukaryotes, not just insects.

### 2.3. SAR

Among the sixteen compounds listed in Table 1, only cantharidin **1** and norcantharidin **3** showed any significant larvicidal activities with 100% mortality at concentrations of 500 μg mL^−1^, respectively. Interestingly, the replacement of the anhydride oxygen atom of norcantharidin **3** with nitrogen (compound **5**) resulted in complete loss of activity. Meanwhile, Table 1 showed that the target compounds **6** also lacked any larvicidal activities. Indeed, to result in any larvicidal activity, no modifications of the cyclic anhydride are tolerated, consequently resulting in larvicidal activity. We thought the replacement of the O-atom with N (as N–H and N–R, where R = alkyl of aryl) would furnish us with a better understanding of the relationship between the electronic effect and larvicidal activities; however no larvicidal activity was demonstrated for the modified cyclic anhydride.

The biological activities of the target compounds **7a** to **7m** against *P. xylostella* at a concentration of 500 μg mL^−1^ are summarized in Table 2. To provide structure-activity relationship information about the effect of the aliphatic amide moiety substituent, R, compounds **7a**, **7b** and **7c** were designed to contain −CH_3_, −CH(CH_3_)_2_ and −CH_2_(CH_2_)_2_CH_3_, respectively. Although it is difficult to construct a clear structure-activity relationship from the data shown in Table 2, it can be concluded that the general trend in larvicidal activity for the substituents was −CH_3_ (**7a**) > −CH(CH_3_)_2_ (**7b**) > −CH_2_(CH_2_)_2_CH_3_ (**7c**). For example, compound **7a** (R = −CH_3_, 77%) displayed a significantly higher insecticidal activity than compound **7b** (R = −CH(CH_3_)_2_, 12%) and **7c** (R = −CH_2_(CH_2_)_2_CH_3_, 4%) at 500 μg mL^−1^.

To examine the electronic effect of substituent Y on the aniline ring, the electron-donating substituent –OCH_3_ and electron-withdrawing substituents −CF_3_, –OCF_3_, F and −CO_2_H were introduced. Compounds with electron-withdrawing substituents displayed higher larvicidal activities against *P. xylostella* than compounds with electron-donating substituents, as seen in the comparison of the compounds **7d** (Y = 2′-OCH_3_), **7e** (Y = 2′-F) and **7f** (Y = 2′-NO_2_) of the series with Y at 2′-position on the aniline ring, **7g** (Y = 3′-OCH_3_), **7h** (Y = 3′-F) and **7i** (Y = 3′-CF_3_) of the series with Y at 3′-position on the aniline ring, and **7j** (Y = 4′-OCH_3_), **7k** (Y = 4′-CO_2_H), **7l** (Y = 4′-F) and **7m** (Y = 4′-OCF_3_) of the series with Y at 4′-position on the aniline ring. These observations revealed that substitution patterns on the aniline ring have an important influence on the larvicidal activity. Compounds with electron-withdrawing substituents showed excellent larvicidal activities against *P. xylostella*, while compounds with electron-donating substituents display lower larvicidal activity.

In addition, as shown in Table 2, compound **2**, **4** and **7f** were the most active compounds. All of their larvicidal activities against *P. xylostella* at 500 μg mL^−1^ were 100% after 48 h, while the larvicidal activity of the parent compounds **1** and **2** were 100% at the same concentration after 48 h as shown in Table 1. These results indicated that compounds **2**, **4** and **7f** displayed comparable larvicidal activity with their corresponding lead compound against *P. xylostella* at 500 μg mL^−1^. Therefore, we carried out further insecticidal activity assay for compounds **2**, **4** and **7f**, and cantharidin **1** and norcantharidin **2** were used as a control to make a judgment on the larvicidal potency of these compounds. As shown in Table 3, it was found that the LC_50_ values of compounds **4** and **7f** against *P. xylostella* were 0.70 mM and 0.43 mM respectively, while that of the parent compound norcantharidin was 0.84 mM. Moreover, the LC_50_ of compounds **2** was the same as that of its parent compound cantharidin **1** with value of 0.06 mM.

The larvicidal activity values of anhydride analogs in Table 1 were compared with those of their ring-opened dicarboxylic acid partners in Table 2. The anhydride analogs indicated no any larvicidal activity, except **1** and **3**, as mentioned above. However, their ring-opened dicarboxylic acid partners all suggested various larvicidal activities. On the one hand, the larvicidal activities of cantharidin **1** and norcantharidin **3** were equal to that of their ring-opened partners, compounds **2** and **4** with LC_50_ values of 0.06 mM, 0.84 mM, 0.06 mM, and 0.70 mM in Table 3, respectively. And the larvicidal activities of cantharidin **1** and norcantharidin **3** appeared to be the results of the spontaneous hydrolyzing process to ring-opened compounds **2** and **4**. On the other hand, compared with **1** and **3**, compounds **6** were so stable that they could not be hydrolyzed to their ring-opened partners naturally, which leads to the absence of larvicidal activity. Therefore, we conjectured that the active form of cantharidin and its derivatives is the dicarboxylic acid analog, a ring-opened compound.

## 3. Experimental Section

### 3.1. General Experimental Procedures

Melting points of all compounds were determined on a WRS-2 apparatus (Shanghai Precision & Scientific Instrument Co. Ltd., Shanghai, China) and are uncorrected. ^1^H NMR and ^13^C NMR spectra were obtained using a Bruker AVANCE-500 MHz spectrometer in CDCl_3_ or DMSO solution with tetramethylsilane as the internal standard. Chemical shift values (δ) were given in parts per million (ppm). High-resolution mass spectrometry (HRMS) data were obtained on a Bruker micrOTOF-Q II instrument.

Cantharidin **1** was isolated from *Mylabris phalerata* (Chinese blister beetle), which was bought from the Chinese herbal medicine market in Xian, China. Norcantharidin **3** was purchased from Alfa Aesar Chemical Co. Ltd. (Haverhill, MA, USA). All primary amine reagents were of analytical reagent grade. Solvents were dried and purified using standard techniques immediately before use. Silica gel for TLC and CC was obtained from Qingdao Haiyang Chemical Co. Ltd. (Qingdao, China).

### 3.2. Synthetic Procedures

#### 3.2.1. General Synthetic Procedure for Compound **2**

To a solution of cantharidin **1** (390 mg, 2 mmol) in water (10 mL) was dropwise added a solution of sodium hydroxide (180 mg, 4.5 mmol) in water (5 mL). This was stirred at 100 °C for 2 h. After the mixture had cooled to room temperature, a solution of hydrogen chloride (4.5 mmol) in water (9 mL) was added dropwise to the mixture and stirred overnight at room temperature. The white slurry was filtered and the cake washed with dichloromethane. The crude solid was recrystallized from methanol to give **2** as white solid (400 mg, 93%). mp: 232–233 °C. ^1^H NMR (500 MHz, D_2_O) δ: 1.03 (s, 6H, H-9, 10), 1.44 (d, *J* = 8.51 Hz, 2H, H-1, 2, *exo*), 1.76 (d, *J* = 7.88 Hz, 2H, H-1, 2, *endo*), 4.53 (br, s, 2H, H-3, 6). ^13^C NMR (125 MHz, D_2_O) δ: 17.26 (C-9, C-10), 23.98 (C-1, C-2), 58.45 (C-4, C-5), 84.29 (C-3, C-6), 183.94 (C-7, C-8). HR-MS (ESI): *m*/*z* calcd for C_10_H_13_O_5_ ([M−1]^−^) 213.0763, found 213.0773.

#### 3.2.2. General Synthetic Procedure for Compound **4**

A mixture of norcantharidin **3** (1.0 g, 5.95 mmol) and water (15 mL) was refluxed for 8 h. After the mixture had cooled to room temperature, the reaction was concentrated *in vacuo* and filtered. The resulting precipitate was recrystallized from methanol to obtain compound **4** as white solid (960 mg, 87%). mp: 137–138 °C. ^1^H NMR (500 MHz, DMSO-d_6_) δ: 1.48–1.60 (m, 4H, H-1, 2), 2.92 (s, 2H, H-4, 5), 4.67 (d, *J* = 2.21 Hz, 2H, H-3, 6), 12.05 (br, s, 2H, H-9, 10). ^13^C NMR (125 MHz, DMSO-d_6_) δ: 29.08 (C-1, C-2), 52.03 (C-4, C-5), 78.20 (C-3, C-6), 172.89 (C-7, C-8). HR-MS (ESI): *m*/*z* calcd for C_8_H_9_O_5_ ([M−1]^−^) 185.0450, found 185.0455.

#### 3.2.3. General Synthetic Procedure for Compound **5**

A mixture of norcantharidin **3** (1.0 g, 5.95 mmol), carbamide (220 mg, 3.57 mmol) and DMF (1.4 mL) was heated at 130–135 °C. After the mixture had melted completely, it was allowed to react at 160–165 °C. When the reaction was completed according to TLC analysis, the mixture cooled to room temperature. After filtration, the crude solid was recrystallized from EtOAc/Hexane (1:1, *v*/*v*) resulting in product **5** as yellow solid (920 mg, 88.5%). mp: 186–188 °C. ^1^H NMR (500 MHz, CDCl_3_) δ: 1.60 (d, *J* = 7.57 Hz, 2H, H-1, 2, *exo*), 1.87 (d, *J* = 7.57 Hz, 2H, H-1, 2, *endo*), 2.93 (br, s, 2H, H-4, 5), 4.92 (br, s, 2H, H-3, 6), 8.33 (br, s, 1H, H-9). ^13^C NMR (125 MHz, CDCl_3_) δ: 28.52 (C-1, C-2), 51.36 (C-4, C-5), 79.12 (C-3, C-6), 177.23 (C-7, C-8). HR-MS (ESI): *m*/*z* calcd for C_8_H_9_NO_3_Na ([M+Na]^+^) 190.0480, found 190.0474.

#### 3.2.4. General Synthetic Procedure for the Target Compounds **6** (**a**–**m**)

Primary amine (1 equiv, 5.59 mmol) was added to a stirred mixture of norcantharidin **3** (1.0 g, 5.95 mmol), triethylamine (1.5 mL) and toluene (15 mL). The reaction was carried out at reflux temperature, and the progress was monitored by TLC. After completion, the mixture was moved to *vacuo*. The residue was either recrystallized from EtOAc/Hexane (1:1, *v*/*v*) or purified by column chromatography on a silica gel using EtOAc and hexane (EtOAc/Hexane, 2:5, *v*/*v*) as the eluent to afford the target compounds **6**. The yields, physical properties, ^1^H NMR, ^13^C NMR, and HR-MS of the target compounds **6** were as follows:

*Data for****6***-***a***: white solid; yield, 93%; mp: 132–133 °C; ^1^H NMR (500 MHz, CDCl_3_) δ: 1.58–1.64 (m, 2H, H-1, 2, *exo*), 1.83–1.90 (m, 2H, H-1, 2, *endo*), 2.89 (s, 2H, H-4, 5), 2.96 (s, 3H, H-1′), 4.88 (br, s, 2H, H-3, 6); ^13^C NMR (125 MHz, CDCl_3_) δ: 25.07 (C-1′), 28.59 (C-1, C-2), 50.03 (C-4, C-5), 79.00 (C-3, C-6), 177.26 (C-7, C-8); HR-MS (ESI): *m*/*z* calcd for C_9_H_11_NO_3_Na ([M+Na]^+^) 204.0637, found 204.0633.

*Data for****6***-***b***: white solid; yield, 71%; mp: 141–142 °C; ^1^H NMR (500 MHz, CDCl3) δ: 1.36 (d, *J* = 6.94 Hz, 6H, H-2′, 3′), 1.56–1.62 (m, 2H, H-1, 2, *exo*), 1.80–1.88 (m, 2H, H-1, 2, *endo*), 2.79 (s, 2H, H-4, 5), 4.32 (dt, *J* = 13.87, 6.94 Hz, 1H, H-1′), 4.82–4.89 (m, 2H, H-3, 6); 13C NMR (125 MHz, CDCl3) δ: 19.16 (s, 2C, C-2′, C-3′), 28.63 (s, 2C, C-1, C-2), 43.99 (s, 1C, C-1′), 49.58 (s, 2C, C-4, C-5), 79.18 (s, 2C, C-3, C-6), 177.27 (s, 2C, C-7, C-8); HR-MS (ESI): *m*/*z* calcd for C_11_H_15_NO_3_Na ([M+Na]^+^) 232.0950, found 232.0953.

*Data for****6***-***c***: white solid; yield, 89%; mp: 80–81 °C; ^1^H NMR (500 MHz, CDCl_3_) δ: 0.91 (t, *J* = 7.25 Hz, 3H, H-4′), 1.29 (sxt, *J* = 7.31 Hz, 2H, H-3′), 1.52 (quin, *J* = 7.41 Hz, 2H, H-2′), 1.60 (d, *J* = 7.25 Hz, 2H, H-1, 2, *exo*), 1.86 (d, *J* = 7.88 Hz, 2H, H-1, 2, *endo*), 2.86 (s, 2H, H-4, 5), 3.46 (t, *J* = 7.25 Hz, 2H, H-1′), 4.87 (br, s, 2H, H-3, 6); ^13^C NMR (125 MHz, CDCl_3_) δ: 13.62 (C-4′), 19.94 (C-3′), 28.61 (C-1, C-2), 29.65 (C-2′), 38.88 (C-1′), 49.87 (C-4, C-5), 79.07 (C-3, C-6), 177.28 (C-7, C-8); HR-MS (ESI): *m*/*z* calcd for C_12_H_17_NO_3_Na ([M+Na]^+^) 246.1106, found 246.1101.

*Data for****6***-***d***: white solid; yield, 71%; mp: 149–150 °C; ^1^H NMR (500 MHz, CDCl_3_) δ: 1.65 (d, *J* = 7.25 Hz, 2H, H-1, 2, *exo*), 1.90 (d, *J* = 7.88 Hz, 2H, H-1, 2, *endo*), 3.00–3.10 (m, 2H, H-4, 5), 3.76–3.82 (m, 3H, H-7′), 4.99 (d, *J* = 2.84 Hz, 2H, H-3, 6), 6.97–7.05 (m, 2H, H-3′, 4′), 7.06–7.17 (m, 1H, H-5′), 7.39 (t, *J* = 7.72 Hz, 1H, H-6′); ^13^C NMR (125 MHz, CDCl_3_) δ: 28.66 (C-1, C-2), 50.41 (C-4, C-5), 55.81 (C-7′), 79.43 (C-3, C-6), 112.09 (C-3′), 112.57 (C-6′), 120.98 (C-1′), 129.32 (C-5′), 130.84 (C-4′), 154.57 (C-2′), 176.28 (C-7, C-8); HR-MS (ESI): *m*/*z* calcd for C_15_H_15_NO_4_Na ([M+Na]^+^) 296.0899, found 296.1001.

*Data for****6***-***e***: white solid; yield, 71%; mp: 139–140 °C; ^1^H NMR (500 MHz, CDCl_3_) δ: 1.62–1.70 (m, 2H, H-1, 2, *exo*), 1.87–1.95 (m, 2H, H-1, 2, *endo*), 3.09 (br, s, 2H, H-4, 5), 5.00 (br, s, 2H, H-3, 6), 7.18–7.22 (m, 1H, H-4′), 7.23 (br, s, 2H, H-3′, 5′), 7.37–7.45 (m, 1H, H-6′); ^13^C NMR (125 MHz, CDCl_3_) δ: 28.64 (s, 2C, C-1, C-2), 50.39 (s, 2C, C-4, C-5), 79.51 (s, 2C, C-3, C-6), 119.65 (s, 1C, C-3′), 124.67 (br, s, 1C, C-5′), 128.24 (s, 1C, C-1′), 129.42 (br. s, 1C, C-4′), 131.01 (s, 1C, C-6′), 156.29 (s, 1C, C-2′), 175.54 (s, 2C, C-7, C-8); HR-MS (ESI): *m*/*z* calcd for C_14_H_12_FNO_3_Na ([M+Na]^+^) 284.0699, found 284.0691.

*Data for****6***-***f***: white solid; yield, 52%; mp: 179–181 °C; ^1^H NMR (500 MHz, CDCl_3_) δ: 1.68 (d, *J* = 7.25 Hz, 2H, H-1, 2, *exo*), 1.92 (d, *J* = 7.88 Hz, 2H, H-1, 2, *endo*), 3.11–3.21 (m, 2H, H-4, 5), 4.96–5.06 (m, 2H, H-3, 6), 7.41 (d, *J* = 7.57 Hz, 1H, H-4′), 7.61 (t, *J* = 7.57 Hz, 1H, H-5′), 7.70–7.80 (m, 1H, H-6′), 8.18 (d, *J* = 8.20 Hz, 1H, H-3′); ^13^C NMR (125 MHz, CDCl_3_) δ: 28.61 (C-1, C-2), 50.59 (C-4, C-5), 79.49 (C-3, C-6), 125.81 (C-6′), 130.19 (C-4′), 130.66 (C-3′), 134.39 (C-5′), 175.47 (C-7, C-8); HR-MS (ESI): *m*/*z* calcd for C_14_H_12_N_2_O_5_Na ([M+Na]^+^) 311.0644, found 311.0623.

*Data for****6***-***g***: white solid; yield, 79%; mp: 147–148 °C; ^1^H NMR (500 MHz, CDCl_3_) δ: 1.62–1.69 (m, 2H, H-1, 2, *exo*), 1.87–1.95 (m, 2H, H-1, 2, *endo*), 3.03 (s, 2H, H-4, 5), 3.81 (s, 3H, H-7′), 5.00 (br, s, 2H, H-3, 6), 6.78 (s, 1H, H-4′), 6.84 (d, *J* = 7.88 Hz, 1H, H-2′), 6.94 (dd, *J* = 8.35, 1.73 Hz, 1H, H-6′), 7.36 (t, *J* = 8.04 Hz, 1H, H-5′); ^13^C NMR (125 MHz, CDCl_3_) δ: 28.68 (C-1, C-2), 50.08 (C-4, C-5), 55.48 (C-7′), 79.56 (C-3, C-6), 112.24 (C-2′), 114.91 (C-4′), 118.81 (C-6′), 129.89 (C-5′), 132.85 (C-1′), 160.14 (C-3′), 176.30 (C-7, C-8); HR-MS (ESI): *m*/*z* calcd for C_15_H_15_NO_4_Na ([M+Na]^+^) 296.0899, found 296.1015.

*Data for****6***-***h***: white solid; yield, 85%; mp: 139–140 °C; ^1^H NMR (500 MHz, CDCl_3_) δ: 1.64–1.70 (m, 2H, H-1, 2, *exo*), 1.87–1.94 (m, 2H, H-1, 2, *endo*), 3.03 (s, 2H, H-4, 5), 4.99 (d, *J* = 2.21 Hz, 2H, H-3, 6), 7.11–7.18 (m, 2H, H-4′, 6′), 7.22–7.29 (m, 2H, H-2′, 5′); ^13^C NMR (125 MHz, CDCl_3_) δ: 28.66 (s, 2C, C-1, C-2), 50.04 (s, 2C, C-4, C-5), 79.57 (s, 2C, C-3, C-6), 116.09 (s, 1C, C-2′), 116.28 (s, 1C, C-4′), 127.75 (s, 1C, C-6′), 128.35 (s, 1C, C-5′), 161.31 (s, 1C, C-1′), 163.29 (s, 1C, C-3′), 176.27 (s, 2C, C-7, C-8); HR-MS (ESI): *m*/*z* calcd for C_14_H_12_FNO_3_Na ([M+Na]^+^) 284.0699, found 284.0692.

*Data for****6***-***i***: white solid; yield, 92%; mp: 197–198 °C; ^1^H NMR (500 MHz, CDCl_3_) δ: 1.65–1.71 (m, 2H, H-1, 2, *exo*), 1.89–1.96 (m, 2H, H-1, 2, *endo*), 3.06 (s, 2H, H-4, 5), 5.01 (d, *J* = 2.21 Hz, 2H, H-3, 6), 7.50 (d, *J* = 7.88 Hz, 1H, H-6′), 7.56–7.62 (m, 2H, H-4′, 5′), 7.63–7.68 (m, 1H, H-2′); ^13^C NMR (125 MHz, CDCl_3_) δ: 28.65 (s, 2C, C-1, C-2), 50.09 (s, 2C, C-4, C-5), 79.64 (s, 2C, C-3, C-6), 123.56 (s, 1C, C-4′), 124.59 (s, 1C, C-7′), 125.44 (s, 1C, C-2′), 129.70 (s, 1C, C-5′), 131.56 (s, 1C, C-3′), 131.82 (s, 1C, C-6′), 132.38 (s, 1C, C-1′), 175.88 (s, 2C, C-7, C-8); HR-MS (ESI): *m*/*z* calcd for C_15_H_12_F_3_NO_3_Na ([M+Na]^+^) 334.0667, found 334.0665.

*Data for****6***-***j***: white solid; yield, 85%; mp: 189–190 °C; ^1^H NMR (500 MHz, CDCl_3_) δ: 1.66 (d, *J* = 7.25 Hz, 2H, H-1, 2, *exo*), 1.90 (d, *J* = 8.20 Hz, 2H, H-1, 2, *endo*), 3.02 (s, 2H, H-4, 5), 3.82 (s, 3H, H-7′), 4.99 (br, s, 2H, 2H, H-3, 6), 6.97 (d, *J* = 8.83 Hz, 2H, H-3′, 5′), 7.17 (d, *J* = 8.83 Hz, 2H, H-2′, 6′); ^13^C NMR (125 MHz, CDCl_3_) δ: 28.69 (C-1, C-2), 40.48 (C-7′), 49.99 (C-4, C-5), 79.47 (C-3, C-6), 112.44 (C-3′, C-5′), 120.25 (C-1′), 127.17 (C-2′, C-6′), 150.57 (C-4′), 176.98 (C-7, C-8); HR-MS (ESI): *m*/*z* calcd for C_15_H_15_NO_4_Na ([M+Na]^+^) 296.0899, found 296.1011.

*Data for****6***-***k***: white solid; yield, %; mp: 270–271 °C; ^1^H NMR (500 MHz, DMSO-d_6_) δ: 1.71 (s, 4H, H-1, 2), 2.94 (q, *J* = 7.25 Hz, 2H, H-4, 5), 4.83 (s, 2H, H-3, 6), 7.31 (d, *J* = 8.20 Hz, 2H, H-2′, 6′), 8.03 (d, *J* = 8.51 Hz, 2H, H-3′, 5′); ^13^C NMR (125 MHz, DMSO-d_6_) δ: 28.48 (s, 2C, C-1, C-2), 50.31 (s, 2C, C-4, C-5), 79.37 (s, 2C, C-3, C-6), 126.87 (s, 2C, C-2′, C-6′), 130.27 (s, 2C, C-3′, C-5′), 133.71 (s, 1C, C-4′), 135.51 (s, 1C, C-1′), 167.83 (s, 1C, C-7′), 177.07 (s, 2C, C-7, C-8); HR-MS (ESI): *m*/*z* calcd for C_30_H_26_N_2_O_10_2Na ([2M+Na]^+^) 597.1531, found 597.1535.

*Data for****6***-***l***: white solid; yield, 71%; mp: 140–141 °C; ^1^H NMR (500 MHz, CDCl_3_) δ: 1.63–1.69 (m, 2H, H-1, 2, *exo*), 1.88–1.94 (m, 2H, H-1, 2, *endo*), 3.03 (s, 2H, H-4, 5), 4.99 (d, *J* = 2.21 Hz, 2H, H-3, 6), 7.10–7.19 (m, 2H, H-3′, 5′), 7.22–7.29 (m, 2H, H-2′, 6′); ^13^C NMR (125 MHz, CDCl_3_) δ: 28.66 (s, 2C, C-1, C-2), 50.04 (s, 2C, C-4, C-5), 79.57 (s, 2C, C-3, C-6), 116.09 (s, 2C, C-3′, C-5′), 127.75 (s, 1C, C-1′), 128.35 (s, 2C, C-2′, C-6′), 163.29 (s, 1C, C-4′), 176.27 (s, 2C, C-7, C-8); HR-MS (ESI): *m*/*z* calcd for C_14_H_12_FNO_3_Na ([M+Na]^+^) 284.0699, found 284.0675.

*Data for****6***-***m***: white solid; yield, 85%; mp: 177–178 °C; ^1^H NMR (500 MHz, CDCl_3_) δ: 1.67 (d, *J* = 7.57 Hz, 2H, H-1, 2, exo), 1.92 (d, *J* = 7.88 Hz, 2H, H-1, 2, endo), 3.05 (s, 2H, H-4, 5), 5.00 (br, s, 2H, H-3, 6), 7.28–7.33 (m, 2H, H-3′, 5′), 7.33–7.38 (m, 2H, H-2′, 6′); ^13^C NMR (125 MHz, CDCl_3_) δ: 28.66 (s, 2C, C-1, C-2) 50.05 (s, 2C, C-4, C-5) 79.62 (s, 2C, C-3. C-6) 119.36 (s, 1C, C-1′) 121.60 (s, 2C, C-3′, C-5′) 127.98 (s, 2C, C-2′, C-6′) 130.22 (s, 1C, C-4′) 148.89 (s, 1C, C-7′) 176.07 (s, 2C, C-7, C-8); HR-MS (ESI): *m*/*z* calcd for C_15_H_12_F_3_NO_4_Na ([M+Na]^+^) 350.0616, found 350.0615.

#### 3.2.5. General Synthetic Procedure for the Target Compounds **7** (**a**–**m**)

A mixture of norcantharidin **3** (1.0 g, 5.95 mmol), primary amine regent (1 equiv, 5.95 mmol) and THF (10 mL) was stirred for several hours at room temperature, and the progress of reaction was monitored by TLC. After norcantharidin disappeared, the reaction was concentrated under reduced pressure and diluted with acetone (100 mL). The resulting precipitate was either recrystallized from methanol or purified by column chromatography (MeOH/CH_2_Cl_2_, 1:4, *v*/*v*) to afford the desired products **7**. The yields, physical properties, ^1^H NMR, ^13^C NMR, and HR-MS of the target compounds **7** were as follows:

*Data for****7***-***a***: white solid; yield, 84%; mp: 144–146 °C; ^1^H NMR (500 MHz, D_2_O) δ: 1.45–1.70 (m, 4H, H-1, 2), 2.57 (s, 3H, H-1′), 2.88–2.95 (m, 2H, H-4, 5), 4.60 (d, *J* = 4.73 Hz, 1H, H-6), 4.79 (d, *J* = 4.41 Hz, 1H, H-3); ^13^C NMR (125 MHz, D_2_O) δ: 25.87 (C-1′), 28.20 (C-1, C-2), 53.84 (C-4), 54.70 (C-5), 78.68 (C-3), 79.39 (C-6), 175.58 (C-8), 177.81 (C-7); HR-MS (ESI): *m*/*z* calcd for C_9_H_12_NO_4_ ([M−1]^−^) 198.0766, found 198.0753.

*Data for****7***-***b***: white solid; yield 74%; mp: 136–137 °C; ^1^H NMR (500 MHz, DMSO-d_6_) δ: 1.02 (dd, *J* = 6.31, 2.52 Hz, 6H, H-2′, 3′), 1.41–1.62 (m, 4H, H-1. 2), 2.75–2.85 (m, 2H, H-4, 5), 3.76 (dq, *J* = 13.52, 6.74 Hz, 1H, H-1′), 4.47 (d, *J* = 4.73 Hz, 1H, H-6), 4.72 (d, *J* = 4.10 Hz, 1H, H-3), 7.16 (d, *J* = 7.88 Hz, 1H, H-9); ^13^C NMR (125 MHz, DMSO-d_6_) δ: 22.67 (s, 1C, C-2′), 22.88 (s, 1C, C-3′), 28.93 (s, 1C, C-1), 29.31 (s, 1C, C-2), 43.13 (s, 1C, C-1′), 52.60 (s, 1C, C-3), 53.60 (s, 1C, C-4), 77.41 (s, 1C, C-3), 79.08 (s, 1C, C-6), 170.20 (s, 1C, C-8), 173.07 (s, 1C, C-7); HR-MS (ESI): *m*/*z* calcd for C_11_H_18_NO_4_ ([M+1]^+^) 228.1236, found 228.1233.

*Data for****7***-***c***: white solid; yield, 83.5%; mp: 139–140 °C; ^1^H NMR (500 MHz, DMSO-d_6_) δ: 0.88 (t, *J* = 7.25 Hz, 3H, H-4′), 1.23–1.32 (m, 2H, H-3′), 1.32–1.40 (m, 2H, H-2′), 1.42–1.63 (m, 4H, H-1, 2), 2.81–2.87 (m, 2H, H-1′), 2.94–3.05 (m, 2H, H-4, 5), 4.48 (d, *J* = 4.41 Hz, 1H, H-6), 4.74 (d, *J* = 4.10 Hz, 1H, H-3), 7.33 (t, *J* = 5.20 Hz, 1H, H-9), 11.89 (br, s, 1H, H-10); ^13^C NMR (125 MHz, DMSO-d_6_) δ: 14.17 (C-4′), 20.06 (C-3′), 28.88 (C-2), 29.34 (C-1), 31.06 (C-2′), 38.65 (C-1′), 51.89 (C-4), 53.56 (C-5), 77.22 (C-3), 79.28 (C-6), 170.76 (C-8), 172.82 (C-7); HR-MS (ESI): *m*/*z* calcd for C_12_H_19_NO_4_Na ([M+Na]^+^) 264.1212, found 264.1213.

*Data for****7***-***d***: white solid; yield, 66%; Mp: 151–152 °C; ^1^H NMR (500 MHz, DMSO-d_6_) δ: 1.52–1.72 (m, 4H, H-1, 2), 3.05–3.17 (m, 2H, H-4, 5), 3.83 (s, 3H, H-7′), 4.71 (d, *J* = 5.04 Hz, 1H, H-6′), 4.89 (d, *J* = 3.47 Hz, 1H, H-3′), 6.86–6.93 (m, 1H, H-5′), 7.02 (d, *J* = 4.10 Hz, 2H, H-3′, 4′), 8.11 (d, *J* = 7.88 Hz, 1H, H-6′), 8.87 (s, 1H, H-9), 12.20 (br, s, 1H, H-10); ^13^C NMR (125 MHz, DMSO-d_6_) δ: 28.96 (C-1, C-2), 52.44 (C-7′), 55.32 (C-4), 56.45 (C-5), 77.52 (C-3), 79.54 (C-6), 111.44 (C-3′), 120.32 (C-6′), 120.79 (C-5′), 123.84 (C-3′), 128.31 (C-4′), 148.84 (C-2′), 169.95 (C-8), 172.61 (C-7); HR-MS (ESI): *m*/*z* calcd for C_15_H_17_NO_5_Na ([M+Na]^+^) 314.1004, found 314.1008.

*Data for****7***-***e***: white solid; yield, 85%; mp: 114–115 °C; ^1^H NMR (500 MHz, DMSO-d_6_) δ: 1.53–1.68 (m, 4H, H-1, 2), 3.04 (d, *J* = 9.46 Hz, 1H, H-4), 3.21 (d, *J* = 9.77 Hz, 1H, H-5), 4.61–4.77 (m, 1H, H-6), 4.78–4.93 (m, 1H, H-3), 7.06–7.19 (m, 2H, H-3′, 5′), 7.20–7.28 (m, 1H, H-4′), 8.02 (t, *J* = 7.72 Hz, 1H, H-6′), 9.28 (s, 1H, H-9), 12.13 (br, s, 1H, H-10); ^13^C NMR (125 MHz, DMSO-d_6_) δ: 28.92 (s, 1C, C-1), 29.11 (s, 1C, C-2), 52.28 (s, 1C, C-4), 54.13 (s, 1C, C-5), 77.54 (s, 1C, C-3), 79.36 (s, 1C, C-6), 115.53 (s, 1C, C-3′), 123.24 (s, 1C, C-1′), 124.83 (s, 1C, 6′), 126.95 (s, 1C, 4′), 152.24 (s, 1C, C-5′), 154.17 (s, 1C, C-2′), 170.22 (s, 1C, C-8), 172.71 (s, 1C, C-7); HR-MS (ESI): *m*/*z* calcd for C_14_H_14_FNO_4_Na ([M+Na]^+^) 302.0805, found 302.0807.

*Data for****7***-***f***: yellow solid; yield, 47%; mp: 160–161 °C; ^1^H NMR (500 MHz, DMSO-d_6_) δ: 1.46–1.72 (m, 4H, H-1, 2), 2.98–3.12 (m, 2H, H-4, 5), 4.63–4.85 (m, 2H, H-3, 6), 7.60 (t, *J* = 8.20 Hz, 1H, H-4′), 7.82 (d, *J* = 7.88 Hz, 1H, H-5′), 7.90 (d, *J* = 7.88 Hz, 1H, H-6′), 8.65 (br, s, 1H, H-3′), 10.28 (br, s, 1H, H-9), 12.02–12.09 (m, 1H, H-10); ^13^C NMR (125 MHz, DMSO-d_6_) δ: 28.91 (C-2), 29.44 (C-1), 52.24 (C-4), 53.82 (C-5), 77.49 (C-3), 79.00 (C-6), 113.68 (C-6′), 117.96 (C-4′), 125.55 (C-3′), 130.53 (C-5′), 140.91 (C-1′), 148.42 (C-2′), 170.61 (C-8), 172.63 (C-7); HR-MS (ESI): *m*/*z* calcd for C_14_H_14_N_2_O_6_Na 329.0750, found 329.0754.

*Data for****7***-***g***: white solid; yield, 73%; mp: 165–166 °C; ^1^H NMR (500 MHz, DMSO-d_6_) δ: 1.47–1.70 (m, 4H, H-1, 2), 2.95 (d, *J* = 9.46 Hz, 1H, H-4), 3.07 (d, *J* = 9.77 Hz, 1H, H-5), 4.64 (d, *J* = 4.41 Hz, 1H, H-6), 4.80 (d, *J* = 3.78 Hz, 1H, H-3), 6.61 (dd, *J* = 8.20, 1.89 Hz, 1H, H-4′), 7.05 (d, *J* = 8.20 Hz, 1H, H-6′), 7.15–7.22 (m, 1 H, H-5′), 7.29 (s, 1H, H-2′), 9.68 (s, 1H, H-9), 11.99 (br, s, 1H, H-10); ^13^C NMR (125 MHz, DMSO-d_6_) δ: 28.91 (C-2), 29.46 (C-1), 52.05 (C-7′), 54.03 (C-4), 55.42 (C-5), 77.34 (C-3), 79.23 (C-6), 105.36 (C-6′), 108.97 (C-2′), 111.89 (C-4′), 129.81 (C-5′), 140.95 (C-1′), 159.95 (C-3′), 169.83 (C-8), 172.72 (C-7); HR-MS (ESI): *m*/*z* calcd for C_15_H_17_NO_5_Na ([M+Na]^+^) 314.1004, found 314.0983.

*Data for****7***-***h***: white solid; yield, 86%; mp: 163–164 °C; ^1^H NMR (500 MHz, DMSO-d_6_) δ: 1.41–1.71 (m, 4H, H-1, 2), 2.97 (d, *J* = 9.46 Hz, 1H, H-4), 3.07 (d, *J* = 9.46 Hz, 1H, H-5), 4.66 (d, *J* = 4.41 Hz, 1H, H-6), 4.80 (d, *J* = 3.78 Hz, 1H, H-3), 6.85 (td, *J* = 8.43, 2.36 Hz, 1H, H-4′), 7.22 (d, *J* = 8.20 Hz, 1H, H-6′), 7.29–7.36 (m, 1H, H-5′), 7.57 (d, *J* = 11.66 Hz, 1H, H-2′), 9.95 (s, 1H, H-9) 12.01 (s, 1H, H-10); ^13^C NMR (125 MHz, DMSO-d_6_) δ: 28.89 (s, 1C, C-1), 29.44 (s, 1C, C-2), 52.17 (s, 1C, C-4), 53.89 (s, 1C, C-5), 77.38 (s, 1C, C-3), 79.07 (s, 1C, C-6), 106.28 (s, 1C, C-4′), 109.72 (s, 1C, C-2′), 115.31 (s, 1C, C-6′), 130.62 (s, 1C, C-5′), 141.46 (s, 1C, C-1′), 161.65 (s, 1C, C-3′), 170.19 (s, 1C, C-8), 172.64 (s, 1C, C-7); HR-MS (ESI): *m*/*z* calcd for C_14_H_14_FNO_4_Na ([M+Na]^+^) 302.0805, found 302.0811.

*Data for****7***-***i***: white solid; yield, 49%; mp: 174–176 °C; ^1^H NMR (500 MHz, DMSO-d_6_) δ: 1.43–1.76 (m, 4H, H-1, 2), 2.99 (d, *J* = 9.46 Hz, 1H, H-4), 3.09 (d, *J* = 9.46 Hz, 1H, H-5), 4.69 (d, *J* = 2.84 Hz, 1H, H-6), 4.80 (br, s, 1H, H-3), 7.38 (d, *J* = 7.57 Hz, 1H, H-5′), 7.54 (t, *J* = 7.88 Hz, 1H, H-4′), 7.68 (d, *J* = 7.88 Hz, 1H, H-6′), 8.12 (br, s, 1H, H-2′), 10.11 (s, 1H, H-9), 12.04 (s, 1H, H-10); ^13^C NMR (125 MHz, DMSO-d_6_) δ: 28.90 (s, 1C, C-1), 29.43 (s, 1C, C-2), 52.19 (s, 1C, C-4), 53.85 (s, 1C, C-5), 77.43 (s, 1C, C-3), 79.04 (s, 1C, C-6), 115.67 (s, 1C, C-4′), 119.70 (s, 1C, C-7′), 123.08 (s, 1C, C-6′), 123.56 (s, 1C, C-2′), 125.72 (s, 1C, C-5′), 130.29 (s, 1C, C-3′), 140.51 (s, 1C, C-1′), 170.43 (s, 1C, C-8), 172.67 (s, 1C, C-7); HR-MS (ESI): *m*/*z* calcd for C_15_H_14_F_3_NO_4_Na ([M+Na]^+^) 352.0773, found 352.0775.

*Data for****7***-***j***: white solid; yield, 71%; mp: 167–168 °C; ^1^H NMR (500 MHz, DMSO-d_6_) δ: 1.46–1.69 (m, 4H, H-1, 2), 2.94 (d, *J* = 9.77 Hz, 1H, H-4), 3.03 (d, *J* = 9.46 Hz, 1H, H-5), 4.63 (d, *J* = 4.10 Hz, 1H, 1H, H-6), 4.79 (d, *J* = 3.78 Hz, 1H, 1H, H-3), 6.87 (d, *J* = 8.83 Hz, 2H, H-3′, 5′), 7.44 (d, *J* = 8.83 Hz, 2 H, H-2′, 6′), 9.51 (s, 1H, H-9), 11.93 (br, s, 1H, H-10); ^13^C NMR (125 MHz, DMSO-d_6_) δ: 28.89 (C-2), 29.47 (C-1), 52.07 (C-7′), 53.87 (C-4), 55.64 (C-5), 77.29 (C-3), 79.20 (C-6), 114.20 (C-3′, C-5′), 121.24 (C-2′, C-6′), 132.91 (C-1′), 155.54 (C-4′), 169.33 (C-8), 172.76 (C-7); HR-MS (ESI): *m*/*z* calcd for C_15_H_17_NO_5_Na ([M+Na]^+^) 314.1004, found 314.1025.

*Data for****7***-***k***: white solid; yield, 44%; mp: 269–270 °C; ^1^H NMR (500 MHz, DMSO-d_6_) δ: 1.52–1.64 (m, 4H, H-1, 2), 2.98 (d, *J* = 9.46 Hz, 1H, H-4), 3.11 (d, *J* = 9.77 Hz, 1H, H-5), 4.68 (d, *J* = 4.41 Hz, 1H, H-6), 4.80 (d, *J* = 3.78 Hz, 1H, H-3), 7.66 (d, *J* = 8.83 Hz, 2H, H-2′, 6′), 7.89 (d, *J* = 8.83 Hz, 2H, H-3′, 5′), 10.05 (s, 1H, H-9), 12.35 (br, s, 2H, H-10); ^13^C NMR (125 MHz, DMSO-d_6_) δ: 28.91 (s, 1C, C-1) 29.44 (s, 1C, C-2) 52.20 (s, 1C, C-4) 53.97 (s, 1C, C-5) 77.42 (s, 1C, C-3) 79.11 (s, 1C, C-6) 118.80 (s, 2C, C-2′, C-6′) 125.29 (s, 1C, C-4′) 130.78 (s, 2C, C-3′, C-5′) 143.69 (m, 1C, C-1′) 167.32 (m, 1C, C-7′) 170.32 (s, 1C, C-8) 172.65 (s, 1C, C-7); HR-MS (ESI): *m*/*z* calcd for C_15_H_15_NO_6_Na ([M+Na]^+^) 328.0797, found 328.0796.

*Data for****7***-***l***: white solid; yield, 78%; mp: 157–159 °C; ^1^H NMR (500 MHz, DMSO-d_6_) δ: 1.45–1.69 (m, 4H, H-1, 2), 2.93–2.98 (m, 1H, H-4), 3.02–3.08 (m, 1H, H-5), 4.65 (d, *J* = 4.10 Hz, 1H, H-6), 4.79 (d, *J* = 3.78 Hz, 1H, H-3), 7.13 (t, *J* = 8.83 Hz, 2H, H-2′, 6′), 7.55 (dd, *J* = 8.83, 5.04 Hz, 2H, H-3′, 5′), 9.73 (s, 1H, H-8), 11.98 (br, s, 1H, H-7); ^13^C NMR (125 MHz, DMSO-d_6_) δ: 28.90 (s, 1C, C-1), 29.46 (s, 1C, C-2), 52.15 (s, 1C, C-4), 53.83 (s, 1C, C-5), 77.34 (s, 1C, C-3), 79.10 (s, 2C, C-6), 115.49 (s, 2C, C-3′, C-5′), 121.37 (s, 2C, C-2′, C-6′), 136.13 (s, 1C, C-1′), 157.37 (s, 1C, C-4′), 169.71 (s, 1C, C-8), 172.68 (s, 1C, C-7); HR-MS (ESI): *m*/*z* calcd for C_14_H_14_FNO_4_Na ([M+Na]^+^) 302.0805, found 302.0813.

*Data for****7***-***m***: yellow solid; yield, 47%; mp: 170–171 °C; ^1^H NMR (500 MHz, DMSO-d_6_) δ: 1.46–1.69 (m, 4H, H-1, 2), 2.95–3.00 (m, 1H, H-4), 3.07 (d, *J* = 9.77 Hz, 1H, H-5), 4.67 (d, *J* = 4.41 Hz, 1H, H-6), 4.80 (d, *J* = 3.78 Hz, 1H, H-3), 7.30 (d, *J* = 8.83 Hz, 2H, H-3′, 5′), 7.65 (d, *J* = 8.83 Hz, 2H, H-2′, 6′), 9.91 (s, 1H, H-9), 12.01 (s, 1H, H-10); ^13^C NMR (125 MHz, DMSO-d_6_) δ: 28.84 (m, 1C, C-1), 29.44 (m, 1C, C-2), 52.17 (m, 1C, C-4), 53.89 (m, 1C, C-5), 77.34 (m, 1C, C-3), 79.07 (m, 1C, C-6), 120.88 (s, 2C, C-3′, C-5′), 121.65 (s, 1C, C-7′), 121.97 (s, 2C, C-2′, C-6′), 138.91 (m, 1C, C-1′), 143.80 (m, 1C, C-4′), 169.85 (m, 1C, C-8), 172.47 (m, 1C, C-7); HR-MS (ESI): *m*/*z* calcd for C_15_H_14_F_3_NO_5_Na ([M+Na]^+^) 368.0722, found 368.0732.

### 3.3. Bioassay

*Plutella xylostella* were continuously maintained in our laboratory without exposure to any insecticide at 25 ± 2 °C, 50% RH with a photoperiod of 16L:8D. The larvae were reared on pakchoi seedlings.

Compounds were dissolved in component solvent (water:acetone:dimethyl sulfoxide = 20:19:1, *v*/*v*) containing 0.5% Tween-80 to the different concentrations. The bioactivities of all compounds against the early third instar larvae of *P. xylostella* were tested using cotyledons of pakchoi seedlings. The seedlings were treated by firstly dipping them in the test solutions for about 5 s, then placing cotyledons slanting over a blotting paper to drain superfluous fluid and to dry the test solution for about 25 min at room temperature.

For each bioassay, ten treated leaves were put on a wet filter paper (Ø 9 cm) paved in a Petri plate (Ø 9 cm) to keep fresh, and ten larvae were released to the plate after a starvation for 4 h, and kept in a growth cabinet under the same conditions the larvae were reared. Each sample was repeated four times and mortality was recorded after 48 h. A component solvent containing 0.5% Tween-80 was used as control. Bioassay data were polled and analyzed by standard probit analysis [23], using Abbott’s correction for control mortality [24], and the toxicity was ascertained by estimating the median lethal dose (LD_50_, killing 50%) of each compound.

## 4. Conclusions

In summary, two series of cantharidin analogs (compounds **6** and compounds **7**) containing alkyl and aryl groups at 9-position were designed and synthesized. Their structures were confirmed by ^1^H NMR, ^13^C NMR and HRMS. The larvicidal activities of cantharidin and its derivatives against the pre-third-instar *P. xylostella* were evaluated. All of the cyclic compounds except cantharidin **1** and norcantharidin **3** lacked any larvicidal activity. The ring-opened cantharidin derivatives demonstrated different activities, and compound **7f** showed the highest larvicidal activity with LC_50_ value of 0.43 mM. Structure-activity relationship study indicated that the form of compound (cyclic or ring-opened) or their ability to hydrolyze facilely is the key to determine whether it exhibits larvicidal activity. Meanwhile, this revealed that the improvement of insecticidal activity required a reasonable combination of both aliphatic amide and aromatic amide moieties, and the type of substituent Y on the aniline ring was critical.

## Figures and Tables

**Table 1:** Structures and larvicidal activities against *P. xylostella* of compounds **5**, **6**, **1** and **3**.

Compounds	R	Concentration (μg mL^−1^)	Mortality (%)
5	H	500	0
6a	−CH_3_	500	0
6b	−CH(CH_3_)_2_	500	0
6c	−CH_2_(CH_2_)_2_CH_3_	500	0
6d	2′-OMePh	500	0
6e	2′-FPh	500	0
6f	2′-NO_2_Ph	500	0
6g	3′-OMePh	500	0
6h	3′-FPh	500	0
6i	3′-CF_3_Ph	500	0
6j	4′-OMePh	500	0
6k	4′-CO_2_HPh	500	0
6l	4′-FPh	500	0
6m	4′-OCF_3_Ph	500	0
Cantharidin 1	-	500	100
Norcantharidin 3	-	500	100

**Table 2:** Structures and larvicidal activities against *P. xylostella* of compounds **7**, **2** and **4**.

Compounds	R	Concentration (μg mL^−1^)	Mortality (%)
7a	−CH_3_	500	77
7b	−CH(CH_3_)_2_	500	12
7c	−CH_2_(CH_2_)_2_CH_3_	500	4
7d	2′-OMePh	500	25
7e	2′-FPh	500	60
7f	2′-NO_2_Ph	500	100
7g	3′-OMePh	500	24
7h	3′-FPh	500	41
7i	3′-CF_3_Ph	500	32
7j	4′-OMePh	500	11
7k	4′-CO_2_HPh	500	97
7l	4′-FPh	500	38
7m	4′-OCF_3_Ph	500	19
2	-	500	100
4	-	500	100

**Table 3:** Insecticidal activities against *P. xylostella* of compounds **1**–**4** and **7f**.

Compounds	*y* = a + b*x*	*r*	LC_50_ (95% CI) (mM)	LC_90_ (95% CI) (mM)
Cantharidin 1	*y* = 9.4287 + 3.6777*x*	0.99	0.06 (0.06–0.07)	0.14 (0.11–0.17)
2	*y* = 9.6322 + 3.8866*x*	0.99	0.06 (0.05–0.07)	0.14 (0.11–0.18)
Norcantharidin 3	*y* = 5.4052 + 5.1887*x*	0.97	0.84 (0.75–0.93)	1.48 (0.94–1.97)
4	*y* = 5.8547 + 5.4057*x*	0.98	0.70 (0.64–0.77)	1.20 (0.94–1.52)
7f	*y* = 6.8918 + 5.2902*x*	0.99	0.43 (0.39–0.48)	0.76 (0.59–0.99)
